# Elevated CRB3 expression suppresses breast cancer stemness by inhibiting β‐catenin signalling to restore tamoxifen sensitivity

**DOI:** 10.1111/jcmm.13619

**Published:** 2018-03-30

**Authors:** Pingping Li, Chen Feng, He Chen, Yina Jiang, Fang Cao, Jie Liu, Peijun Liu

**Affiliations:** ^1^ Center for Translational Medicine The First Affiliated Hospital of Xi'an Jiaotong University Xi'an China; ^2^ Key Laboratory for Tumor Precision Medicine of Shaanxi Province The First Affiliated Hospital of Xi'an Jiaotong University Xi'an China; ^3^ Department of Oncology Shaanxi Provincial Corps Hospital Xi'an China; ^4^ Department of Pathology The First Affiliated Hospital of Xi'an Jiaotong University Xi'an China

**Keywords:** β‐catenin, breast cancer, cancer stem cells, CRB3, tamoxifen

## Abstract

Tamoxifen is a first‐line drug for hormone therapy (HT) in oestrogen receptor‐positive breast cancer patients. However, 20% to 30% of those patients are resistant to tamoxifen treatment. Cancer stem cells (CSCs) have been implicated as one of the mechanisms responsible for tamoxifen resistance. Our previous study indicated that decreased expression of the CRB3 gene confers stem cell characteristics to breast cancer cells. In the current investigation, we found that most of the breast cancer patient tissues resistant to tamoxifen were negative for CRB3 protein and positive for β‐catenin protein, in contrast to their matched primary tumours by immunohistochemical analysis. Furthermore, expression of CRB3 mRNA and protein was low, while expression of β‐catenin mRNA and protein was high in tamoxifen resistance cells (LCC2 and T47D TamR) contrast to their corresponding cell lines MCF7 and T47D. Similarly, CRB3 overexpression markedly restored the tamoxifen sensitivity of TamR cells by the MTT viability assay. Finally, we found that CRB3 suppressed the stemness of TamR cells by inhibiting β‐catenin signalling, which may in turn lead to a decrease in the breast cancer cell population. Furthermore, these findings indicate that CRB3 is an important regulator for breast cancer stemness, which is associated with tamoxifen resistance.

## 1 INTRODUCTION

Breast cancer is the most commonly diagnosed cancer among Chinese women, accounting for 15% of the total cancer incidence in women, even more so in middle‐aged and young women; in the United States, breast cancer accounts for 30% of total cancer incidence in women.^1^, ^2^ Clinical cancer statistics show that it is the leading cause of cancer death among Chinese women under the age of 45 and that age‐standardized incidence ratios and mortality have increased significantly.^1^, ^2^ As breast cancer is endocrine‐dependent, long‐term stimulation of oestrogen through binding of the oestrogen receptor (ER) plays a critical role in its occurrence and progression. As the majority of breast cancer patients are ERα(+), hormone therapy (HT), including treatment using ER antagonists or anti‐oestrogen therapeutic agents, appears to be one of the important treatment options leading to long‐term survival of breast cancer patients.^3^, ^4^ Long‐term clinical observations have revealed that HT has several advantages such as low toxicity, easy treatment and comparable therapeutic efficacy over chemotherapy. Currently, there are three main types of drugs used in HT: selective oestrogen receptor modulators (SERMs), aromatase inhibitors (AIs) and selective oestrogen receptor down‐regulators (SERDs).^5^

Tamoxifen, one of the SERMs, is a first‐line treatment drug for HT in oestrogen‐positive breast cancer. It competes with oestrogen for ER in vivo and in turn inhibits the biological functions of oestrogen, and reduces mortality and recurrence rates of breast cancer, as demonstrated by the 5‐year survival rate of ER(+) patients of breast cancer rising to 85% in recent years.^6^ However, approximately 20%~30% of ER(+) breast cancer patients acquired tamoxifen resistance.^6^ As tamoxifen resistance drastically enhances malignancy, metastasis and mortality of breast cancer, it has become a limitation in the therapeutic application of tamoxifen and a barrier that ultimately affects the prognosis of breast cancer patients. Therefore, it is important to identify the major molecular targets that lead to tamoxifen resistance, molecularly predict tamoxifen resistance, assess more effective treatment strategies and design a potential rational regimen for combination therapy.

CRB3, the human orthologue of CRB, is a tumour suppressor in mammalian epithelial cells. Loss of CRB3 was observed in mice which were initially injected with a non‐tumorigenic kidney cell line and later developed tumours spontaneously through in vivo selection, and re‐expressing CRB3 restored cell‐cell junctions, cell polarity and contact inhibition.^7^ CRB3 is also able to relay cell density information to regulate proliferation of mouse mammary cells.^8^ Ben Margolis et al reported that transmembrane protein CRB3 also localized to a membrane compartment at the mitotic spindle poles and played a crucial role in cell division via unique interactions with importin β‐1.^9^ Our previous study showed that CRB3 expression was associated with poor prognosis in 136 pairs of human ccRCC (clear cell renal cell carcinoma) and adjacent normal kidney tissues by Immunohistochemistry.^10^ Altogether, decreased expression of the CRB3 gene activated the Wnt signalling pathway, resulting in the non‐tumorigenic, immortalized breast epithelial cell line MCF 10A acquiring cancer stem cell (CSC) properties.^11^ It has been postulated that tamoxifen resistance is attributed to the expansion of cancer stem cells.^12^, ^13^, ^14^ Thus, we in this study sought to investigate whether CRB3 could restore tamoxifen sensitivity of breast cancer cells by suppressing CSC properties via blocking the Wnt signalling pathway.

## 2 MATERIALS AND METHODS

### 2.1 Analysis of immunohistochemistry (IHC)

IHC was performed on paraffin sections from nine metastatic tissues resistant to tamoxifen and matched primary tumours (PT) of breast cancer patients (Table 1). These patients were admitted to the First Affiliated Hospital of Xi'an Jiaotong University, and had developed relapse (local recurrence or metastasis) under regular adjuvant hormone therapy (tamoxifen 20 mg/d). Tumour specimens were processed and embedded into paraffin. After the slides were incubated with the blocking solution (goat serum) for 15 minutes, they were placed in 0.01 mol/L sodium citrate in a microwave oven (pH 6.0, 100 W, 6 minutes; 50 W, 13 minutes) for antigen retrieval. Subsequently, the slides were incubated with the primary antibodies—CRB3 (1:200, HPA013835, Sigma‐Aldrich, Louis, MO, USA) and β‐catenin (1:200, #8480, Cell Signaling, Beverly, MA, USA) at 4°C overnight. The slides were stained using the 3,3′‐diaminobenzidine (DAB) and counterstained with haematoxylin, and were observed under Leica microscope (SCN 400; Mannheim, Germany). Each IHC image was read and scored by a pathologist twice on three different microscopic fields each time. The pathologist was blinded to the group allocation.

**Table 1:** Clinicopathologic characteristics of metastatic breast cancer tissues resistant to tamoxifen and matched primary tumours

Sample ID	Site of Metastasis	TAM therapy received
TamR‐Met1	PT—left breast (9/2009)	9/2009‐3/2011
	Recurrence of left chest wall and diffuse lung metastasis (3/2011)	
TamR‐Met2	PT—left breast (5/2008)	5/2008‐3/2011
	Recurrence of left chest wall and right breast (3/2011)	
TamR‐Met3	PT—right breast (2/2009)	2/2009‐4/2011
	Recurrence of right chest wall (4/2011)	
TamR‐Met4	PT—left breast (1/2007)	1/2007‐3/2009
	Recurrence of left chest wall (1/2011)	Toremifene 3/2009‐1/2011
TamR‐Met5	PT—right breast (1/2009)	1/2009‐3/2011
	Recurrence of bone (3/2011)	
TamR‐Met6	PT—left breast (7/2007)	7/2007‐1/2009
	Recurrence of left clavicular lymph node (1/2010)	Letrozole 1/2009‐1/2010
TamR‐Met7	PT—left breast (9/2005)	4/2007‐2/2011
	Recurrence of left chest wall (2/2011)	
TamR‐Met8	PT—right breast (10/2013)	10/2013‐8/2015
	Recurrence of bone (8/2015)	
TamR‐Met9	PT—right breast (2/2014)	2/2014‐9/2015
	Recurrence of right chest wall (9/2015)	

The IHC staining intensity was scored as 0 (negative), 1 (weakly positive), 2 (moderately positive) or 3 (strongly positive). The extent of staining was defined as the percentage of stained cells per field and scored as 0 (<10%), 1 (10%‐40%), 2 (40%‐70%) or 3 (>70%). The staining score for each field was calculated as the combination of the intensity and the extent of the staining.

### 2.2 Cell culture, transfection and infection

Human breast cancer cells T47D were obtained from the Institute of Basic Medical Sciences Chinese Academy of Medical Sciences & School of Basic Medicine Peking Union Medical College. Human breast cancer cells MCF7 were given by Jianmin Zhang (Roswell Park Cancer Institute, Buffalo, New York, USA). MCF7 cells were cultured in DMEM (Hyclone, Logan, UT, USA) supplemented with 10% foetal bovine serum (FBS) (Hyclone). T47D cells were cultured in RPMI 1640 (Hyclone) supplemented with 10% FBS (Hyclone) and 0.2 units/mL of bovine insulin (#I‐1882, Sigma‐Aldrich).^15^ The tamoxifen‐resistant (TamR) sublines (LCC2 or T47D TamR) were derived from MCF7 or T47D by continuous exposure to tamoxifen as previously described.^16^ LCC2 and T47D TamR cells were maintained in phenol red‐free DMEM containing 5% charcoal‐dextran–stripped FBS supplemented with 1 μmol/L 4‐hydroxytamoxifen in ethanol (H7904, Sigma‐Aldrich). Prior to the assays, all cells were cultured in phenol red‐free DMEM containing 5% charcoal‐dextran–stripped FBS for 1 week. All cells were maintained at 37°C in a humidified atmosphere containing 5% CO_2_. All cell lines had only been passaged within 3 months, and the cell lines were characterized by the Genetic Testing Biotechnology Corporation (Suzhou, China) using short tandem repeat markers.

CRB3‐overexpressing lentivirus, CRB3‐knockdown lentivirus and CRB3‐knockdown siRNA were purchased from GenePharma Company (Shanghai, China). CRB3‐overexpressing lentivirus (or knockdown lentivirus) was transduced into LCC2, or T47D TamR cells (or MCF7 cells) in the presence of 5 μg/mL of polybrene. After 72 hours, cells were maintained in medium containing 2 μg/mL of puromycin. CRB3‐knockdown siRNA sequences (5′‐3′) were as follows: CRB3‐1 sense, AUG AGA AUA GCA CUG UUU UTT; CRB3‐2 sense, UGG CAC UGU UGG UGC GGA ATT. One‐hundred and‐sixty‐six pmol of siRNA and 17 μL of Lipofectamine™ 2000 (Invitrogen, Life Technologies, Shanghai, China) were diluted separately in 250 μL of Opti‐MEM (Gibco, Carlsbad, CA, USA), respectively. The diluted siRNA and Lipofectamine™ 2000 reagents were mixed and incubated at room temperature for 5 minutes. The siRNA‐lipid complex was then added to cells that had been grown to 30%‐50% confluence in 6‐cm plates for 48 hours.

### 2.3 Real‐time PCR

The primers were designed by TaKaRa. Measurements were performed in triplicate and normalized to GAPDH levels. Primer pairs used in real‐time PCR are listed in Table 2.

**Table 2:** Primer pairs used in real‐time PCR

Gene	Primer sequences (5′‐3′)
*GAPDH*	
F	CTC CTC CAC CTT TGA CGC TG
R	TCC TCT TGT GCT CTT GCT GG
*CRB3*	
F	CTT CTG CAA ATG AGA ATA GCA CTG
R	GAA GAC CAC GAT GAT AGC AGT GA
β*‐catenin*	
F	CAT TCA GCA GAA GGT CCG AGT G
R	TGG CTG AGC TGG CTG TTG A
*CD44*	
F	GCA TTG CAG TCA ACA GTC GAA GA
R	CCT TGT TCA CCA AAT GCA CCA
*cMyc*	
F	GGA GGC TAT TCT GCC CAT TTG
R	CGA GGT CAT AGT TCC TGT TGG TG
*NANOG*	
F	CCT GTG ATT TGT GGG CCT GA
R	CTC TGC AGA AGT GGG TTG TTT G

### 2.4 Western blot analysis

Whole‐cell lysates were prepared in modified RIPA buffer; proteins were separated by SDS/PAGE and transferred onto PVDF membranes, which were incubated with primary antibodies. HRP‐conjugated secondary antibodies (Cell Signaling) were used. Chemiluminescent signals were detected using ECL Plus (Millipore, Temecula, CA, USA). CRB3 antibody (1:500, #292449) and cMyc antibody (1:500, #764) were purchased from Santa Cruz (Dallas, Texas); OCT4 antibody (1:1000, WL1005a) was purchased from Wanleibio (Shenyang, China); β‐catenin antibody (1:1000, #8480), NANOG antibody (1:1000, #4903), CD44 (1:1000, #3570) were purchased from Cell Signaling; and GAPDH antibody (1:10 000, HRP‐60004) was obtained from Proteintech (Wuhan, China).

### 2.5 Cell viability assay

The cells were seeded in 48‐well plates at a density of 10^4^ cells/well. One day post‐seeding, various concentrations of tamoxifen were added for 24, 48 or 72 hour. DMSO was used as a control. At the end of the treatment, 5 mg/mL of MTT reagent in 1/10 of the medium volume was added to the medium and incubated for 4 hours at 37°C. After removing the medium, 200 μL of MTT solvent (DMSO) was added to each well for 15 minutes, and the optical density (OD) values were read using a microplate reader (PerkinElmer, Waltham, MA, USA) (λ = 490 nm). Each experiment was repeated three times. All OD values in experimental groups were normalized by converting them to the percentage of the mean value in control group. The tamoxifen resistance factor (RF) was calculated as the IC50 of TamR cells divided by IC50 of parental cells.

### 2.6 Analysis of immunofluorescence

Cells were fixed in 4% paraformaldehyde, permeabilized with 0.3% Triton X‐100 and stained with the following primary antibodies: CRB3 (1:200, HPA013835, Sigma‐Aldrich,) and β‐catenin (1:200, #8480, Cell Signaling) overnight at 4°C. Goat anti‐rabbit Alexa Fluor 488‐conjugated (A11008) secondary antibodies were purchased from Thermo Fisher Scientific (San Jose, CA, USA).

### 2.7 Fluorescence‐activated cell sorting (FACS) analysis

One million cells were stained with APC‐conjugated CD44 antibody (#103008, Biolegend) and PE‐conjugated CD24 antibody (#311118, Biolegend) to detect CD44^high^/CD24^low^ subpopulations.

The ALDEFLUOR kit (#01700; Stem Cell Technologies) was used to detect intracellular aldehyde dehydrogenase (ALDH) enzyme activity.

For carboxyfluorescein diacetate succinimidyl ester (CFSE) analysis, the cells were stained with 2.5 μmol/L CFSE at 37°C for 30 minutes. Cells were passed through a 35‐μm filter, pelleted, washed in 1 × PBS + 0.5% FBS and counted.

### 2.8 Mammosphere formation assay

Mammosphere formation assay was performed by plating 1 × 10^4^ cells in serum‐free DMEM/F12 media (Gibco) supplemented with EGF (20 ng/mL, Peprotech, Rocky Hill, NJ, USA) and B27 (2%, Invitrogen) into ultra‐low attachment 6‐well plates (#3471, Corning, Corning, NY, USA). Mammospheres were allowed to grow for 6 days. Total mammospheres greater than 100 μm in diameter were counted.

### 2.9 Animals, xenotransplantation and treatments

All animal experiments were approved by the Institutional Animal Care and Use Committee of Xi'an Jiaotong University. A total of 2.5 × 10^6^ cells resuspended in 100 μL of PBS were injected subcutaneously into the mammary fat pads of 6‐week‐old female SCID/Beige mice (Laboratory Animal Center of Xi'an Jiaotong University, China). Tamoxifen (5 mg/kg in peanut oil) was administered daily by gavage as previously described.^17^ Tumour volume was calculated using the following formula: (long axis × short axis^2^)/2.

### 2.10 Statistical analysis

Statistical analyses were performed in GraphPad Prism, version 7.00. The statistical significance between two groups was compared by unpaired *t* test, nonparametric Spearman's correlation or Wilcoxon signed‐rank test; three groups were compared by one‐way anova with Dunnett's multiple comparisons test or two‐way anova with Sidak's multiple comparisons test. All statistical tests were two‐sided. All data were from experiments performed at least three times with similar results. All results are expressed as mean ± SEM (n = 3, *P < .05, **P < .01, ***P < .001, ****P < .0001).

## 3 RESULTS

### 3.1 Association between CRB3 and β‐catenin expression levels correlates with tamoxifen resistance of breast cancer

**Figure 1:**
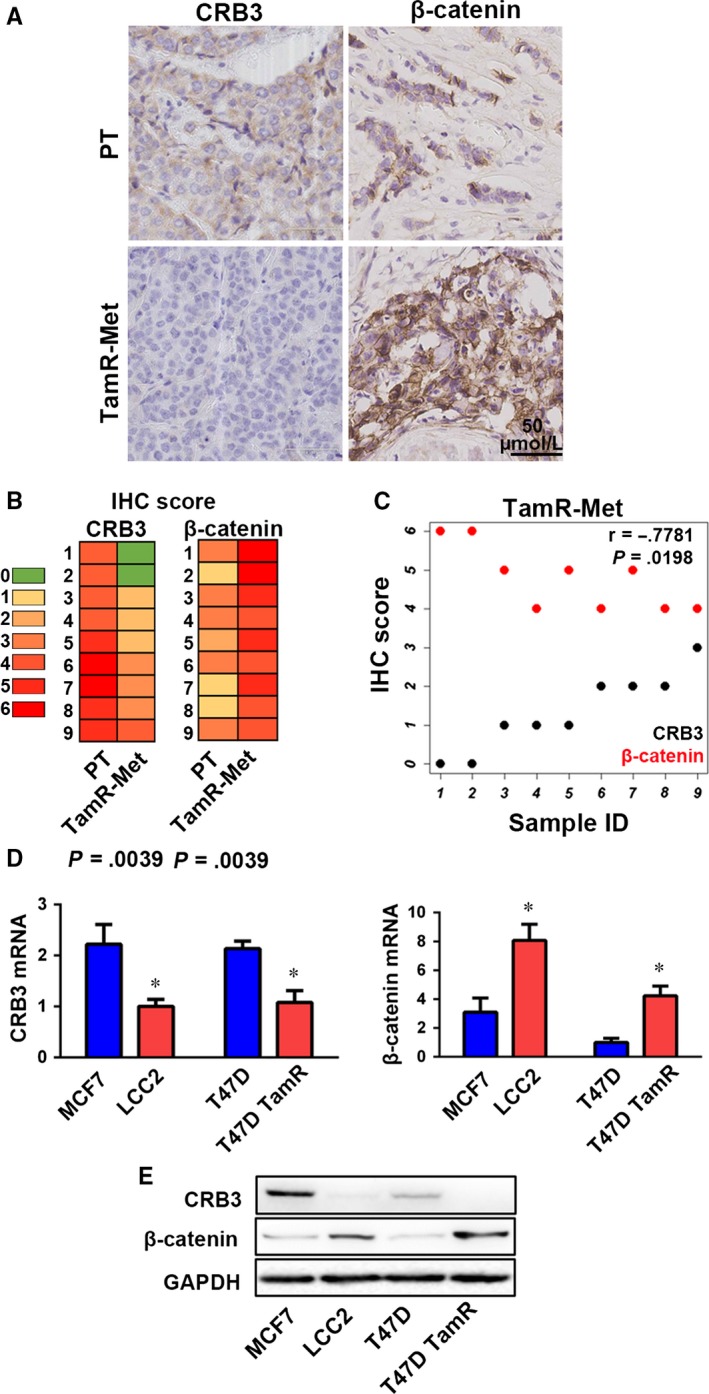
The expression levels of CRB3 and β‐catenin correlate with TAM resistance of breast cancer. (A) Representative images of CRB3 and β‐catenin protein expression in primary tumour (PT) and metastatic tissues from patients who developed tamoxifen resistance and metastasis (TamR‐Met) (Table 1). (B) Representative images of CRB3 and β‐catenin IHC scores (0‐6) in PT and TamR‐Met. (C) CRB3 and β‐catenin are negatively correlated in TamR‐Met. (D) CRB3 and β‐catenin mRNA expression in human breast cancer cells evaluated by real‐time PCR. The expression levels of CRB3 mRNA in other cells were normalized to the one in LCC2 cells, and the expression levels of β‐catenin mRNA in other cells were normalized to the one in T47D cells. (E) Western blot analysis of CRB3 and β‐catenin expression in all cell lines. *P* values were obtained from Wilcoxon signed‐rank test (B), nonparametric Spearman's correlation (C) and unpaired *t* test (D)

To study whether CRB3 expression plays a role in tamoxifen resistance in breast cancer, we examined nine pairs of breast cancer tissues resistant to tamoxifen and matched primary tumours. Immunohistochemistry analysis showed that CRB3 expression was reduced in tamoxifen‐resistant tissues compared to their matched primary tumour counterparts (Figure 1A). As our previous study demonstrated that decreased CRB3 expression leads to activation of the Wnt signalling pathway,^11^ we also examined β‐catenin expression in tamoxifen‐resistant tissues by IHC. As expected, β‐catenin expression was elevated in resistant tissues (Figure 1A). Overall, among the nine pairs of matched tissues, CRB3 protein expression was significantly reduced in metastatic tissues of patients who developed resistance to TAM (*P* = .0039) where β‐catenin protein expression was markedly increased (*P* = .0039) (Figure 1B). We found that the expression levels of CRB3 and β‐catenin proteins showed opposite trend, further demonstrating the correlation between CRB3 and β‐catenin protein expression, and analysis of nonparametric Spearman's correlation showed that they were negatively correlated in tamoxifen resistance and metastasis (TamR‐Met) (*r* = −.7781, *P* = .0198) (Figure 1C). These results suggested that CRB3 down‐regulation accompanied by β‐catenin up‐regulation correlates with tamoxifen resistance of breast cancer.

To investigate whether the observed CRB3 and β‐catenin expression patterns in tamoxifen‐resistant tissues could be also found in vitro, we examined mRNA and protein levels of *CRB3* and β*‐catenin* genes in luminal A breast cancer cells, MCF7, T47D, and corresponding tamoxifen‐resistant cells (LCC2 and T47D TamR). The results showed that TamR cells had the higher expression levels of β*‐catenin* mRNA and protein, and had the lower expression levels of *CRB3* mRNA and protein (Figure 1D and E). These expression patterns strongly suggested that CRB3 and β‐catenin might be involved in tamoxifen resistance of breast cancer.

### 3.2 CRB3 regulates tamoxifen sensitivity of breast cancer cells

**Figure 2:**
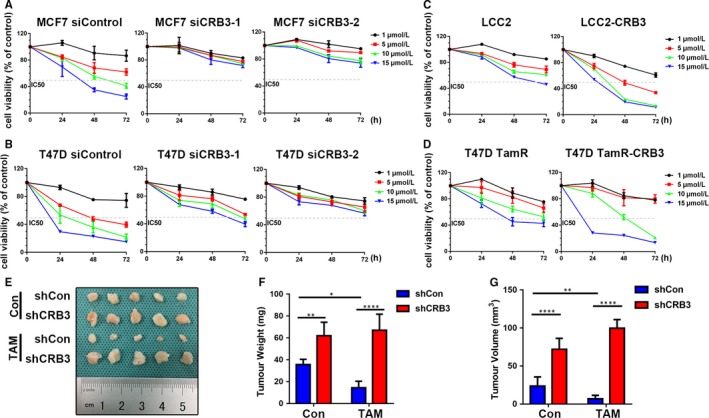
CRB3 regulates tamoxifen sensitivity of breast cancer cells. (A‐D) The cells were treated with various concentrations of tamoxifen. Cell viability was determined using an MTT assay. The percentage of cell viability was calculated from the OD values of the test groups normalized to the control groups. (E‐G) Xenograft tumours were formed by injecting 2.5 × 10^6^ MCF7 cells into the fat pads of SCID/Beige mice. E, Tumours in the different groups are shown. The xenograft weight (F) and size (G) are shown. *P* values were obtained from two‐way ANOVA with Sidak's multiple comparisons test (F and G)

To study the role of CRB3 in tamoxifen sensitivity, we decreased CRB3 expression in MCF7 and T47D cells using siRNA against CRB3 while increasing CRB3 expression in LCC2 and T47D TamR cells using lentivirus‐overexpressing CRB3. The tamoxifen sensitivity of the breast cancer cell lines was assessed by MTT viability assay. Based on the results of the MTT assay at 72 hours, the IC50 for each cell line was as follows: MCF7 6.88 μmol/L; MCF7 siCRB3‐1 843.10 μmol/L; siCRB3‐2 49.28 μmol/L (Figure 2A); T47D 3.01 μmol/L; T47D siCRB3‐1 7.30 μmol/L; T47D siCRB3‐2 69.25 μmol/L (Figure 2B); LCC2 14.76 μmol/L; LCC2‐CRB3 1.77 μmol/L (Figure 2C); T47D TamR 11.15 μmol/L; T47D TamR‐CRB3 7.20 μmol/L (Figure 2D). In addition, the tamoxifen resistance factor (RF) of each cell line was calculated as follows: LCC2‐2.15 and T47D TamR‐3.70. These results showed that CRB3 controls the sensitivity of breast cancer cells towards tamoxifen and that CRB3 overexpression enables to restore tamoxifen sensitivity of tamoxifen‐resistant cells.

To validate the correlation of CRB3 expression with the tamoxifen sensitivity in vivo, xenograft tumour models were established in SCID/Beige mice using MCF7 cells. Seven days after the injection, when the xenograft tumours were palpable, the mice were randomly allocated to tamoxifen (5 mg/kg) by gavage daily. Consistent with the findings of the in vitro experiments, the MCF7 tumours grew more slowly than CRB3‐knockdown MCF7 tumours (Figure 2E). Furthermore, after 2 weeks of treatment with TAM, the tumour sizes and weights decreased remarkably in the mice injected with MCF7 cells compared with those in the CRB3‐knockdown MCF7 cells group (Figure 2F and G), and CRB3‐knockdown MCF7 cells were more resistant to tamoxifen than control MCF7 cells (Figure 2F and G). Taken together, these data support the hypothesis that the changes of CRB3 affect tamoxifen sensitivity.

### 3.3 CRB3 reduces stem cell‐like characteristics of tamoxifen‐resistant cells

**Figure 3:**
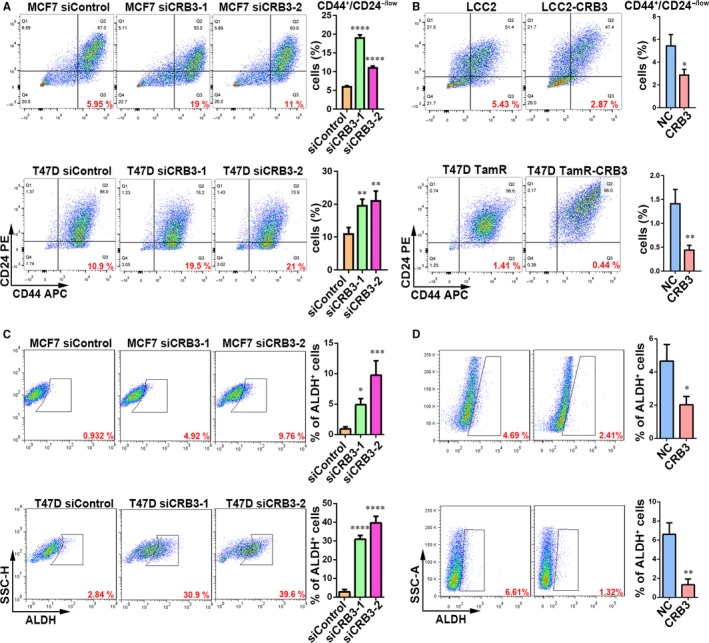
CRB3 reduces CD44^high^/CD24^low^ and ALDH subpopulations of tamoxifen‐resistant cells. (A and B) FACS profiles and quantification of the CD44^high^/CD24^low^ subpopulation. C and D, FACS profiles and quantification of the stem cell marker ALDH subpopulation. *P* values were obtained from one‐way anova with Dunnett's multiple comparisons test (A and C) and unpaired *t* test (B and D)

**Figure 4:**
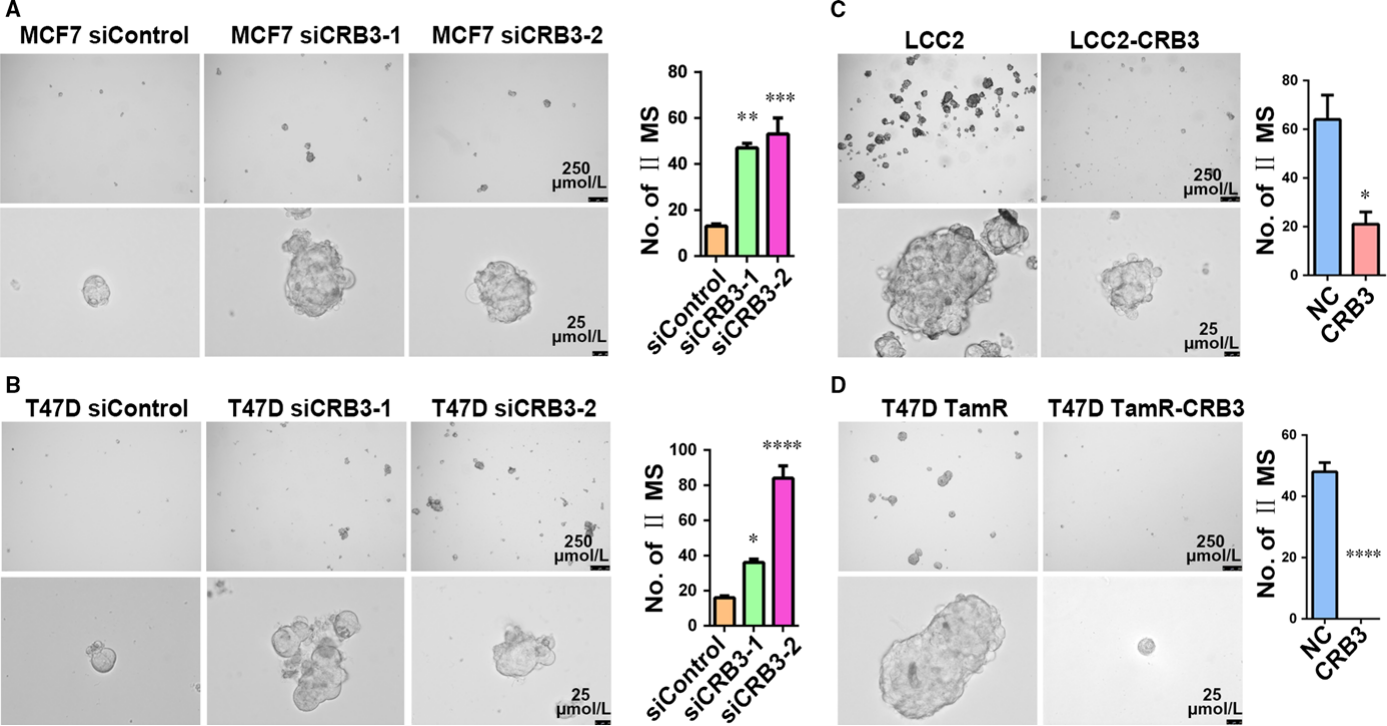
CRB3 reduces formation of mammospheres of tamoxifen‐resistant cells. (A‐D) Representative images and quantification of the formed mammospheres. *P* values were obtained from one‐way anova with Dunnett's multiple comparisons test (A and C) and unpaired *t* test (B and D)

To investigate whether CRB3 regulates the tamoxifen sensitivity of breast cancer cells by suppressing tamoxifen‐resistant cells from acquiring stem cell‐like characteristics, we examined cancer stem cell (CSC) properties of breast cancer cells whose CRB3 expression was altered. CD44^high^/CD24^low^ and ALDH immunophenotypical cells represent a tumour cell population with limited stem cell‐like potential (21, 26). We hence evaluated a tumour cell population with these markers upon changes of CRB3 expression levels. The FACS analysis revealed that the CD44^high^/CD24^low^ (the FMO and isotype control data are shown in Figure S1) and ALDH subpopulations were significantly increased as CRB3 was knocked down and were decreased as CRB3 was overexpressed (Figure 3A‐D). The EGF‐supplemented serum‐free mammosphere formation is a standard assay of CSC self‐renewal.^18^ Similar to the observations in changes of the CD44^high^/CD24^low^ and ALDH subpopulations, CRB3‐knockdown MCF7 and T47D cells displayed increased size and number of mammospheres (Figure 4A and B), while CRB3 overexpression resulted in a decrease in size and number of mammospheres in LCC2 (Figure 4C) and T47D TamR cells (Figure 4D). In aggregate, these results demonstrated that elevated CRB3 expression inhibits stemness of tamoxifen‐resistant breast cancer cells.

### 3.4 CRB3 inactivates β‐catenin in tamoxifen‐resistant breast cancer cells

**Figure 5:**
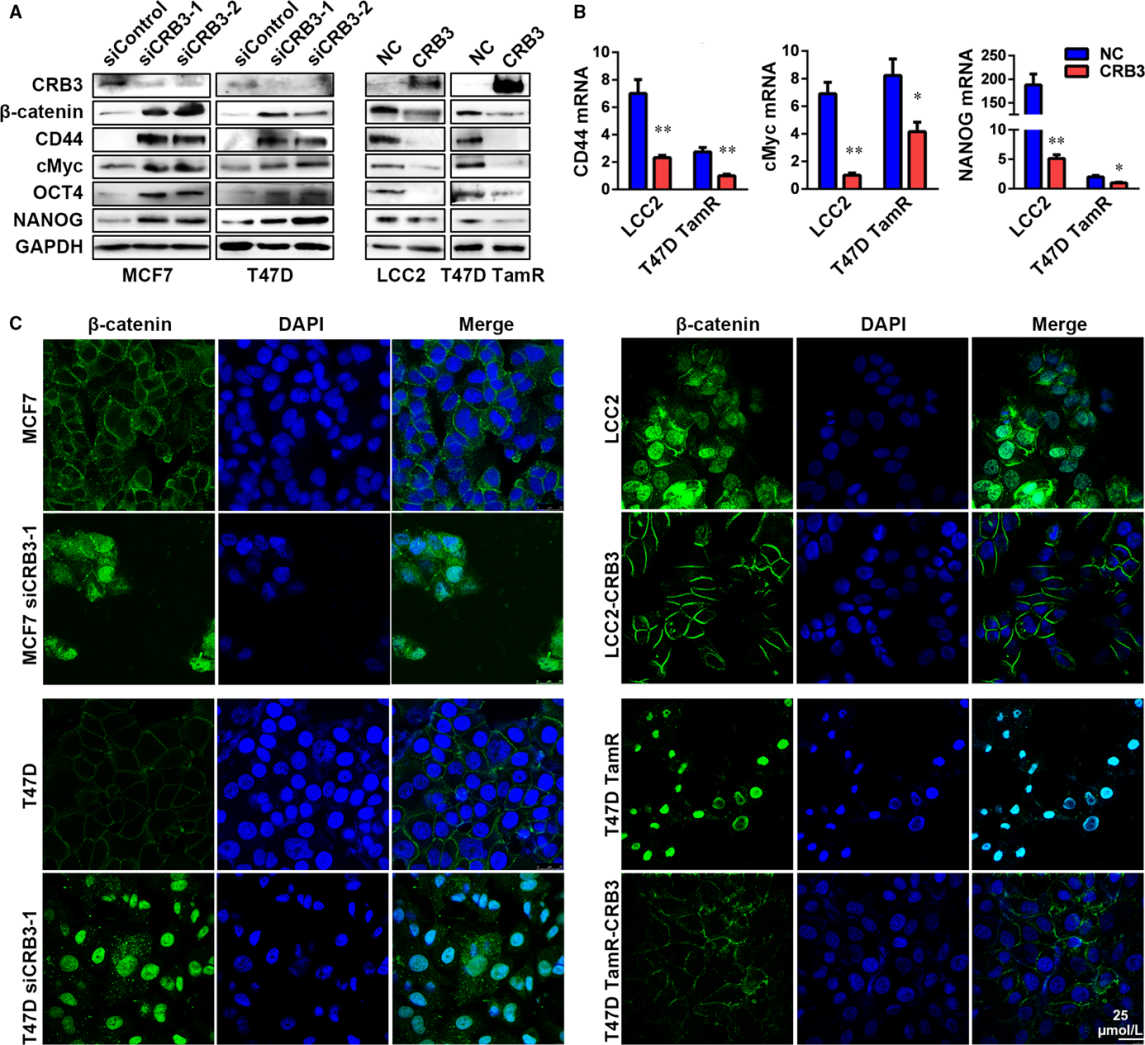
CRB3 inhibits β‐catenin signalling. (A) Western blot analysis of β‐catenin, CD44, cMyc, OCT4 and NANOG expression in CRB3‐overexpressing and CRB3‐knockdown cells. (B) Real‐time PCR analysis of CD44, cMyc and NANOG in CRB3‐overexpressing and CRB3‐knockdown cells. (C) Immunofluorescence analysis of β‐catenin localization is shown. Statistical significance was calculated using unpaired *t* test (B)

The result in Figure 1a suggested that β‐catenin signalling might be active as β‐catenin expression was up‐regulated and it predominantly localized in the nucleus in tamoxifen‐resistant breast cancer tissues. To further examine whether CRB3 downregulation conferred cancer stem cell traits through activation of β‐catenin signalling in tamoxifen‐resistant cells, we assessed whether CRB3 up‐regulation could reduce β‐catenin expression and affect subcellular localization of β‐catenin protein. Consistent with the observations in breast cancer tissues (Figure 1), there was an inverse association between expression level of CRB3 protein and expression levels of β‐catenin protein, β‐catenin downstream factors (CD44 and cMyc) and cancer stem cell‐associated transcription factors (OCT4 and NANOG) (Figure 5A). Real‐time PCR analysis further showed that those expression changes occurred at transcriptional level, as shown by the results that expression of CD44, cMyc and NANOG was declined in LCC2 and T47D TamR cells overexpressing CRB3 (Figure 5B). Finally, β‐catenin was predominantly localized in the cytoplasm and cytomembrane, rather than the nucleus, in CRB3 exogenous expressed tamoxifen‐resistant cells (Figure 5C). Thus, increased CRB3 expression reduced cancer stem cell‐like features of tamoxifen‐resistant cells by inhibiting the β‐catenin signalling pathway.

### 3.5 CRB3 prevents proliferation of tamoxifen‐resistant cells

**Figure 6:**
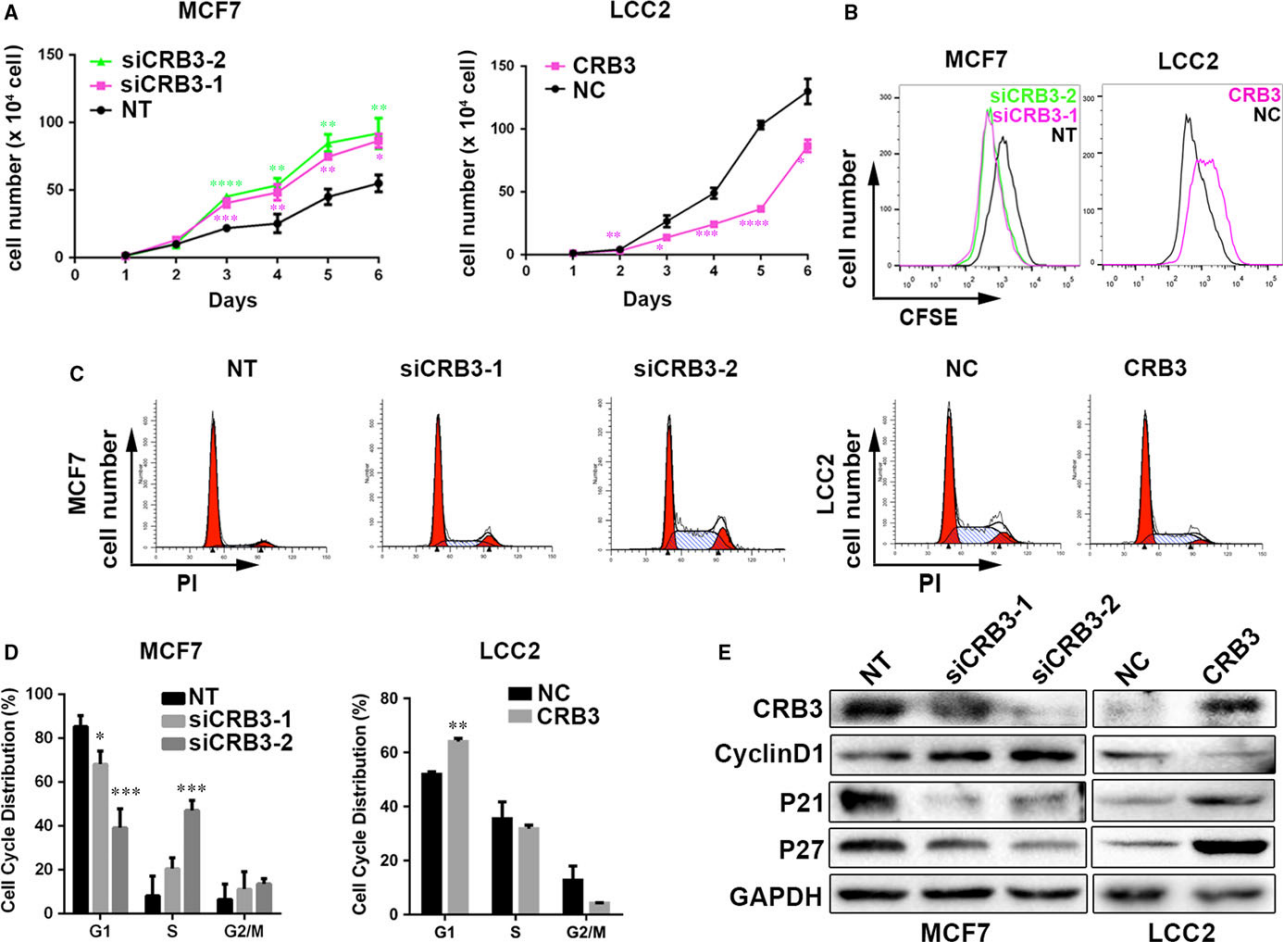
CRB3 inhibits proliferation of tamoxifen‐resistant cells. (A) Growth curves of siCRB3‐transfected MCF7 cells and CRB3‐transfected LCC2 cells. (B) CFSE analysis by FACS. (C) and (D) Flow cytometric analysis of cell cycle. (E) Western blot analysis of regulatory proteins involved in G1/S transition. P values were obtained from one‐way anova with Dunnett's multiple comparisons test and unpaired *t* test (A and D)

As the Wnt/β‐catenin signalling pathway plays a crucial role in regulating cell proliferation,^19^ we examined the effect of CRB3 on proliferation. As shown in Figure 6A, the growth of CRB3–down‐regulated MCF7 cells was increased compared with the parental cells, and CRB3‐overexpressing LCC2 cells exhibited a decreased proliferation rate, which were consistent to cell cycle division analysis by flow cytometry (Figure 6B). CRB3‐knockdown MCF7 cells appeared to mainly accumulate in the S phase, whereas the number of cells in the G1 phase decreased significantly (Figure 6C and D). However, CRB3‐overexpressing LCC2 cells accumulated in the G1 phase. Furthermore, CRB3 up‐regulation decreased cyclin D1 expression but increased p21 and p27 expression, three of which are cell cycle regulatory molecules (Figure 6E). Overall, these results demonstrated that CRB3 up‐regulation inhibited the proliferation of human breast cell lines.

### 3.6 CRB3 affects cell properties through Wnt signalling

**Figure 7:**
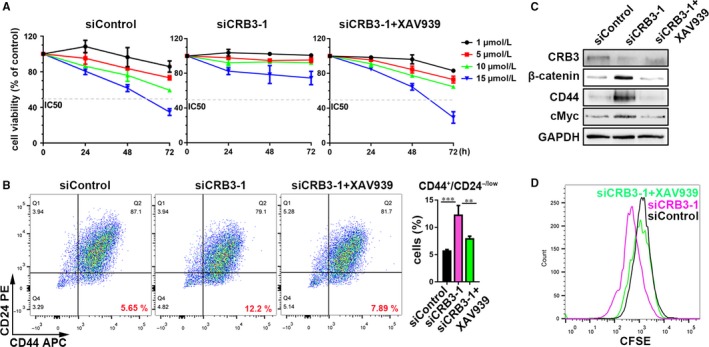
CRB3 affects cell properties through Wnt signalling in MCF7 cells. (A) The MCF7 cells were treated with various concentrations of tamoxifen. Cell viability was determined using an MTT assay. The percentage of cell viability was calculated from the OD values of the test groups normalized to the control groups. (B) FACS profiles and quantification of the CD44^high^/CD24^low^ subpopulation. (C) Western blot analysis of β‐catenin, β‐catenin downstream factors CD44 and cMyc expressions. (D) CFSE analysis by FACS. *P* values were obtained from one‐way anova with Dunnett's multiple comparisons test (B)

Figure 5 results show that CRB3 regulated the Wnt pathway in tamoxifen‐resistant cells. However, it is not known whether CRB3 affects cell properties through Wnt signalling. Therefore, a β‐catenin inhibitor XAV939 (Selleck) was used to inhibit the Wnt pathway in breast cancer cell line MCF7. Compared to CRB3‐knockdown MCF7 cells, XAV939 markedly enhanced tamoxifen sensitivity (Figure 7A). Based on the results of the MTT assay at 72 hours, the IC50 for each cell line was as follows: MCF7 11.06 μmol/L; MCF7 siCRB3‐1 21.79 μmol/L; siCRB3‐1 + XAV939 12.07 μmol/L. We confirmed that β‐catenin inhibitor XAV939 reduced the CD44^high^/CD24^low^ subpopulations (the FMO and isotype control data are shown in Figure S2) in CRB3‐knockdown MCF7 cells (Figure 7B). β‐catenin downstream genes CD44 and cMyc were suppressed by XAV939 in CRB3‐knockdown MCF7 cells (Figure 7C). Furthermore, XAV939 reversed the increase in proliferation of CRB3‐knockdown MCF7 cells (Figure 7D). These results showed that CRB3 affects cell properties through Wnt signalling in tamoxifen‐resistant cells.

## 4 DISCUSSION

Tamoxifen is the first‐line drug for hormone therapy, which was used to treat breast cancer patients. However, tamoxifen resistance has become a roadblock for successful treatment and caused the failure of tamoxifen‐related therapy. Our previous study found that decreased CRB3 expression led to the acquirement of cancer stem cell‐like properties in non‐tumorigenic, immortalized breast epithelial cell line MCF 10A.^11^ Thus, the current study has been suggested that increased expression of cell polarity protein CRB3 might inhibit stemness characteristics of breast cancer that in turn overcomes tamoxifen resistance. We found that CRB3 expression was negatively correlated with tamoxifen resistance in breast cancer patients and that tamoxifen‐resistant cells which overexpress CRB3 were more susceptible to tamoxifen treatment, reduced self‐renewal capability and suppressed the Wnt signalling pathway. These findings demonstrated that CRB3 regulates the stemness properties of breast cancer cells that might affect the response of breast cancer patients to tamoxifen treatment.

A previous study reported that after 18 breast cancer patients were treated with Letrozole for 10‐14 days, expression of a cancer stem cell marker CD44 and number of mammospheres were increased, suggesting that hormone‐resistant cells exhibited more cancerous and stem cell‐like characteristics.^20^ Consistent to those findings, cell population expressing surface antigen CD44^high^/CD24^low^ and ALDH and the ability of forming mammospheres were significantly increased in MCF7 cells that were resistant to tamoxifen.^21^, ^22^, ^23^, ^24^ Furthermore, expression of stem cell factors (NANOG, Sox2 and OCT4) and breast cancer stem cell factor (ABCG2) were increased in tamoxifen resistance cells as demonstrated by DNA methylation and gene expression profiles.^22^ However, the molecular mechanism by which cancer stem cells trigger tamoxifen resistance remains unclear. The results in this study showed that the tamoxifen‐resistant cells had more population displaying stem cell features than parental cells and that enhanced CRB3 expression in those cells reduced the stemness of the breast cancer cells, which ultimately regulates their response to tamoxifen treatment.

It has been demonstrated that the Wnt signalling pathway maintains the undifferentiated state of breast cancer stem cells^25^, ^26^ and has been found activated in approximately 50% of breast cancer.^27^ Surprisingly, treatment of the Wnt signalling pathway agonist Wnt3a in MCF7 cells led to tamoxifen resistance, while treatment of the Wnt signalling pathway inhibitor IWP‐2 restored the sensitivity of MCF7 cells to tamoxifen,^28^ suggesting that an increase in the number of breast cancer stem cells is associated with activation of the Wnt signalling pathway. In addition, inhibition of the long non‐coding RNA UCA1 in MCF7 and T47D cells resulted in reduced activity of the Wnt signalling pathway accompanied by increased tamoxifen sensitivity.^29^ Overall, these studies show that activation of the Wnt signalling pathway plays an important role in the occurrence and development of tamoxifen resistance. Contrary to the previous study that CRB3 knockout mice displayed high levels of cytoplasmic β‐catenin in the intestine, which indicates a low activity of the Wnt signalling pathway,^30^ we found that CRB3 overexpression inhibited the Wnt signalling pathway, which led to decreased expression of downstream genes (CD44 and cMyc) and cancer stem cell factors (OCT4 and NANOG), and that these genes were up‐regulated when cells acquired tamoxifen resistance.

Increased CRB3 expression resumes the response of tamoxifen‐resistant cells to tamoxifen by suppressing breast cancer stem cell‐like features through blocking β‐catenin signalling. While only nine representative tamoxifen‐resistant metastatic tissues and matched primary tumours were examined, and the mechanism whereby CRB3 regulates β‐catenin signalling‐dependent tamoxifen resistance remains to be investigated, we believe that CRB3‐regulated stemness of breast cancer might shed a light in treatment of breast cancer patients who develop resistance to tamoxifen during the course of the treatment.

## CONFLICT OF INTEREST

The authors declare no conflict of interest.

## ETHICS APPROVAL

All procedures performed in studies involving human participants were in accordance with the ethical standards of the institutional and/or national research committee and with the 1964 Helsinki declaration and its later amendments or comparable ethical standards.

## INFORMED CONSENT

Informed consent was obtained from all individual participants included in the study.

## Supporting information


** **

** **
