# Supplementary figures and images for: Elevated CRB3 expression suppresses breast cancer stemness by inhibiting β‐catenin signalling to restore tamoxifen sensitivity
Source: J Cell Mol Med. 2018 Mar 30;22(7):3423–33. doi: 10.1111/jcmm.13619 (PMC6010813; doi:10.1111/jcmm.13619)

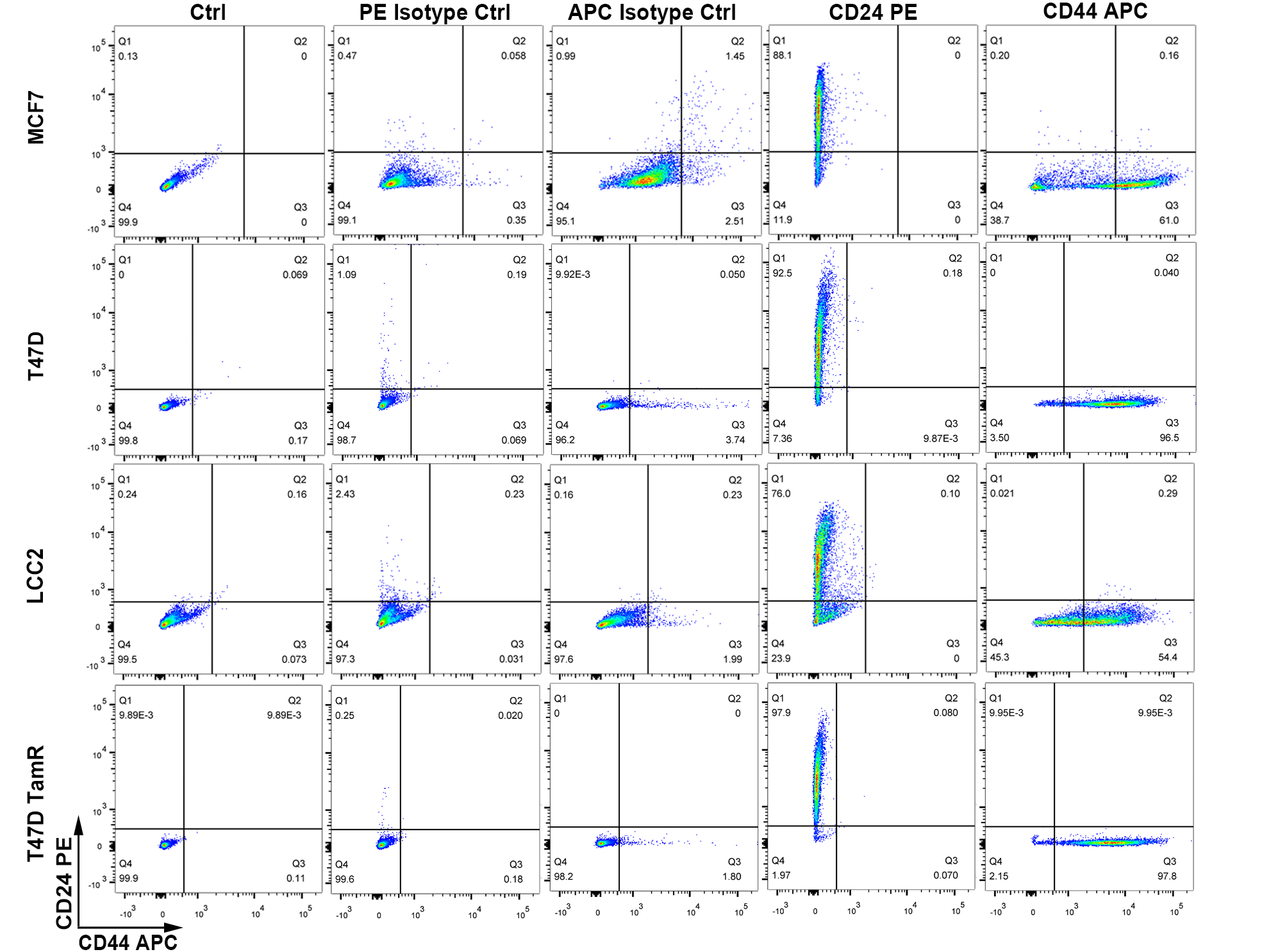

Supplement: Supplementary file 1 [file JCMM-22-3423-s001.tif]

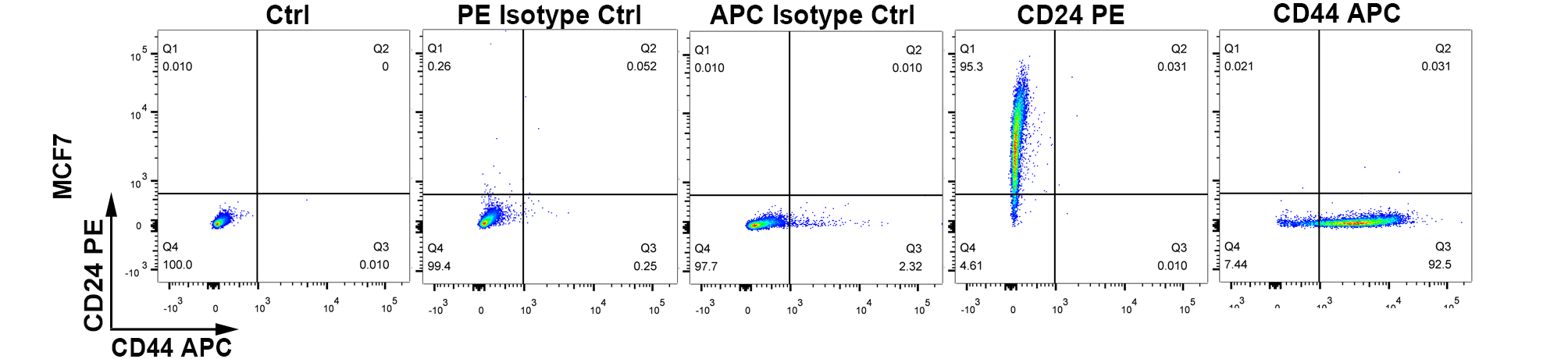

Supplement: Supplementary file 2 [file JCMM-22-3423-s002.tif]
